# User-Friendly Data-Sharing Practices for Fostering Collaboration within a Research Network: Roles of a Vanguard Center for a Community-Based Study

**DOI:** 10.3390/ijerph13010034

**Published:** 2015-12-22

**Authors:** Jae Eun Lee, Jung Hye Sung, M. Edwina Barnett, Keith Norris

**Affiliations:** 1 Research Centers in Minority Institutions Translational Research Network Data Coordinating Center, Mississippi e-Center, Jackson State University, 1230 Raymond Rd., Jackson, MS 39204, USA; m.e.barnett@rtrn.net; 2 Department of Epidemiology and Biostatistics, School of Public Health, Jackson State University, 350 W. Woodrow Wilson Drive Jackson Medical Mall, Jackson, MS 39213, USA; jung.h.lee@jsums.edu; 3 Department of Medicine, David Geffen School of Medicine, UCLA, 911 Broxton Ave, Los Angeles, CA 90024, USA; knorris@ucla.edu

**Keywords:** data-sharing practice, research network, role of coordinating center

## Abstract

Although various attempts have been made to build collaborative cultures for data sharing, their effectiveness is still questionable. The Jackson Heart Study (JHS) Vanguard Center (JHSVC) at the NIH-funded Research Centers in Minority Institutions (RCMI) Translational Research Network (RTRN) Data Coordinating Center (DCC) may be a new concept in that the data are being shared with a research network where a plethora of scientists/researchers are working together to achieve their common goal. This study describes the current practices to share the JHS data through the mechanism of JHSVC. The JHS is the largest single-site cohort study to prospectively investigate the determinants of cardiovascular disease among African-Americans. It has adopted a formal screened access method through a formalized JHSVC mechanism, in which only a qualified scientist(s) can access the data. The role of the DCC was to help RTRN researchers explore hypothesis-driven ideas to enhance the output and impact of JHS data through customized services, such as feasibility tests, data querying, manuscript proposal development and data analyses for publication. DCC has implemented these various programs to facilitate data utility. A total of 300 investigators attended workshops and/or received training booklets. DCC provided two online and five onsite workshops and developed/distributed more than 250 copies of the booklet to help potential data users understand the structure of and access to the data. Information on data use was also provided through the RTRN website. The DCC efforts led to the production of five active manuscript proposals, seven completed publications, 11 presentations and four NIH grant proposals. These outcomes resulted from activities during the first four years; over the last couple of years, there were few new requests. Our study suggested that DCC-customized services enhanced the accessibility of JHS data and their utility by RTRN researchers and helped to achieve the principal goal of JHSVC of scientific productivity. In order to achieve long-term success, the following, but not limited to these, should be addressed in the current data sharing practices: preparation of new promotional strategies in response to changes in technology and users’ needs, collaboration with the Network statisticians, harmonization of the JHS data with the other local-based heart datasets to meet the needs of the potential users from the broader geographical areas, adoption of the RTRN comprehensive data-sharing policy to broaden the variety of research topics and implementation of an ongoing monitoring program to evaluate its success.

## 1. Background

Interdisciplinarity and networking for collaboration are the main characteristics of modern research [1]. As the intellectual and scientific rationale for such interdisciplinary collaboration has been articulated [2], there is an increasing interest and activity in forming research networks [3,4] for fostering efficient collaboration. During the last decade, a variety of research network models have been developed, and enhanced scientific productivity has been reported as a result of these collaborations [5,6,7,8]. However, it is still questionable that substantive and long-term collaboration can be achieved in a research network because of several factors, such as the lack of collaborative tools and/or strategies. Lee and Bozeman [9] suggested that a “collaborative strategy” is one of the significant factors impacting scientific outcome, *i.e.*, publishing productivity. Therefore, it is important to develop effective strategies to exploit the potential benefits of collaboration. Data sharing has been an essential strategy to build partnerships and enhance substantive collaboration within a research network [10,11,12,13].

The benefits of data sharing are well documented [14,15,16,17,18,19]. The sharing of scientific data is beneficial because it facilitates the replication of research results and allows the application of old data in new contexts [20]. From a funding agency perspective, data sharing accelerates scientific progress, meets the tax-payers’ right-to-know, utilizes accumulated research resources efficiently, improves patient outcomes, avoids duplicate research and promotes more efficient management of funds. It also contributes to detecting emerging health threats at the national and/or international level and developing a key preventive tool. The data users can save the time, cost and effort of collecting data. They can decrease the time in moving discoveries from the bench to the bedside and increase the visibility and relevance of research outcomes. The data producers may be reluctant to share the data because of the loss of the opportunity for ownership of the copyright or for proprietary use of the data for their own productivity. However, data producers can also benefit from data sharing. They can increase the chance of their papers being published through cooperative research with outside investigators and can increase the number of citations for their main papers published. The increased citations for research papers may allow investigators to have a more favorable position to obtain funds for the successive research projects, because the number of citations is one of the major criteria to evaluate the quality of published papers and ultimately affects the reputation of the researchers and their institutions.

Extensive attempts have been made to foster a culture of sharing and collaboration in academia by global health and funding agencies [21,22,23]. The National Institutes of Health (NIH) requires applicants seeking $500,000 or more in direct costs in any year of the proposed research to release and share data in a timely manner no later than the acceptance for publication of the main findings from the final dataset [24]. In 2005, the National Science Foundation (NSF) published a manifesto on the importance of collecting and curating datasets for ongoing use. The NSF mandates that all funding proposals include a two-page data management plan describing how the project will conform to the NSF policy on sharing of datasets [25]. Journals, such as Nature or PLoS ONE, increasingly adopt data sharing policies with the objective of promoting public access to data. The private funding agencies, such as the Robert Wood Johnson Foundation, the Bill & Melinda Gates Foundation and Wellcome Trust, have also required applicants to prepare a data-sharing plan. The Bill & Melinda Gates Foundation supports guidance that aims to strengthen data sharing for public health in order to create the right environment for data sharing and to achieve good practices [26].

Academic data sharing is a topic that has received a considerable increase in attention during the last decade. Fetcher *et al.* [20] found from their systematic review of published papers that data sharing-related papers continuously increased since 1988. Despite various efforts for and benefits from data sharing, significant challenges still remain. Van Panhuis *et al*. [27] identified twenty potential barriers from a systematic review and classified them into six categories: technical, motivational, economic, political, legal and ethical. Although it may be difficult to find the effective solutions aimed at any one barrier in isolation because of their interconnectedness, it is documented that structural solutions are readily available (e.g., the funding expertise and cooperation to implement data sharing for the first three barriers and an international dialogue aimed at generating consensus on policies and instruments for data sharing for the last three). Although previous studies emphasized the importance of barriers from the data producers’ side, reuse may depend on the skills and tolerance of potential users (e.g., users’ ability and effort to understand processes and guidelines, skills to handle complex data, tolerability for the time required to resolve IRB issues and acquire the data after approval by data producers, the ability and time to understand data dictionaries and other necessary documentation, *etc.*) [28,29]. Therefore, considering the characteristics of potential re-users in developing the data-sharing program can accelerate the reuse [29]. The Research Centers in Minority Institutions (RCMI) Translational Research Network (RTRN) Data Coordinating Center (DCC) data-sharing practices are designed to resolve the barriers related to data users.

Data sharing is an important ongoing component of a research network to foster collaboration. As part of the endeavor to build collaboration in a research network, the current study aims to leverage lessons learned from the NIH-funded Research Centers in Minority Institutions (RCMI) RTRN DCC to: (1) develop a conceptual approach model to facilitate data use in a research network; (2) describe data-sharing engagement performed by the DCC; and (3) discuss the advantages and challenges of these aggressive strategies in engaging the data-sharing practices. Ultimately, this study is to provide an important template for research networks to effectively share data with network investigators, optimize scientific productivity and foster collaboration among investigators at various levels of training, with different levels of expertise and practicing in environments with different research capacities.

RTRN as the environment of the data-sharing practices: This study will discuss data-sharing practices engaged in RTRN, a research network designed to eliminate health disparities. RTRN is a translational research network comprising 18 RCMI programs that conduct research related to diseases that disproportionately affect the African American, Hispanic, Asian and Pacific Islander and Native American communities. The RTRN was established with the principal goal of creating a framework for effective collaborations within RCMI institutions that include five medical education institutions and 13 biomedical research institutions from 12 different U.S. states. The intent of collaborative relationships was to promote access to information and resources that move the entire research community toward a greater understanding of and access to the tools needed to eradicate health disparities to achieve health equity for all Americans. By linking the researchers through Internet-mediated virtual meeting places or “cyber workspaces”, the RTRN cluster system helps researchers from a broad range of disciplines work together effectively and overcome the barriers imposed by space and time [30]. RTRN’s principal investigator provides direction and leadership for the Network’s three Centers, the Administrative Coordinating Center (ACC), the Research Coordinating Center (RCC) and the Data Coordinating Center (DCC), which offer expertise, services, as well as technical support for the Network’s collaborative initiatives and multi-site research study development and implementation. The types of data shared and the role of a DCC vary across different networks. Ultimately, there is a duty within each network to ensure diligence to the principles of respect for patient/participant confidentiality and privacy, as well as data security. Within RTRN, the DCC provides analytic support and, if requested, secure data storage. The RTRN Ethics and Regulatory Subcommittee also reviews recommendations regarding consent procedures for specific data elements to ensure that IRB approvals include a provision for data sharing when gaining informed consent and the protection of people’s identities by the appropriate de-identification of data where needed, as well as controls for access to data, especially to the linking and use of biological and health data [31,32]. Much of this is coordinated through the Tuskegee University National Center for Bioethics in Research and Health Care, which is the major bioethics resource for the network. This is the nation’s first bioethics center devoted to engaging the sciences, humanities, law and religious faiths in the exploration of the core moral issues that underlie research and medical treatment of vulnerable populations and adhere to the principles of both the Office for Human Research Protections (OHRP) and the United Nations Educational, Scientific and Cultural Organization (UNESCO) Reports and Advices of the International Bioethics Committee. It plays an important role given that many of the Networks’ clinical research activities are directed toward reducing disparities in minority and vulnerable populations. The RTRN Ethics and Regulatory Subcommittee also provides ethical support through a consortium-wide institutional review board (IRB) working group led by two bioethicists, and the working group includes the director of the Tuskegee University National Center for Bioethics in Research and Health Care to provide a direct resource for ethical consultation and other key activities, such as harmonization of clinical research review across institutions [33]. The only projects formally conducted by the center are descriptive studies of the processes, such as the coordination of harmonizing IRBs across the participating institutions.

RCMI investigators have focused on the conduct of research in high-impact health disparity areas, such as cancer, HIV/AIDS and other infectious diseases, cardiovascular disease, neurological disorders and mental health, environmental health and toxicology and drug therapies. This represents a special source of longitudinal data and scientific evidence on the health and living conditions of minority populations at minority institutions, ultimately generating results that translate to the better health of the general public. The details of the RTRN are described elsewhere [34,35].

## 2. Dataset to Be Shared: Jackson Heart Study Data

The Jackson Heart Study (JHS) is a longitudinal epidemiological study designed to investigate the various genotype and phenotype factors that affect high blood pressure, heart disease, stroke, diabetes and other important cardiovascular disease (CVD)-related diseases in African Americans. It is a large, community-based, observational study whose 5301 participants were recruited from the non-institutionalized African-American adults from urban and rural areas of the three counties (Hinds, Madison and Rankin) that make up the Jackson, MS, metropolitan statistical area (MSA). Jackson is the capital of Mississippi, the state with the largest percentage (36.3%) of African Americans in the United States [36].

The sampling frame for the study was a participant in any one of the Atherosclerosis Risk in Communities Study (22%), random (17%), volunteer (30%) or family (31%) samples. Recruitment was limited to non-institutionalized adult African Americans 35–84 years old, except in the family cohort, where those 21–34 years of age were eligible. The baseline examination was completed during 2000–2004; the subsequent examination was done 2005–2008 and 2009–2012. The JHS cohort data included medical history, physical examination, blood/urine analytes and interview questions on areas, such as: physical activity; stress, coping and spirituality; racism and discrimination; socioeconomic position; and access to healthcare [37,38]. The family study focused on hereditary factors, specific genetic variants and gene-environment interactions [38,39]. CVD event data were created through a review of death certificates and hospital records to identify CVD events in the cohort. The data include any CVD event from 10 years since the first JHS contact [40,41].

Data-sharing mechanism of the Jackson Heart Study—JHS Vanguard Center (JHSVC): The JHS has adopted a formal screened access method through the JHSVC mechanism, in which only qualified scientist(s) can access the data. Vanguard Collaborative Centers offer an additional mechanism for sharing Jackson Heart Study (JHS) data to foster collaborations, increase scientific productivity and contribute to achieving the Network’s ultimate goal of eliminating health disparities. The RTRN Vanguard Center may be a new concept in that the data are being shared with a research network where a plethora of scientists/researchers are working together to achieve their common goal. The benefits of Vanguard Centers [42] include: the Vanguard Centers receive a JHS Data Package from the JHS Coordinating Center regardless of the approval of the manuscript proposal. This allows DCC to conduct a feasibility test for data users prior to preparing manuscript development and to save time for obtaining the customized data from JHS data Coordinating Center after approval of manuscript proposal; JHS Vanguard Centers can have access to genetic data on JHS participants, which is usually granted to an institution with expertise and experience in the analysis of genetic data and with approved access to the JHS Genetic database (dbG); and the JHS Coordinating Center will assist in answering queries from the Vanguard Centers regarding the JHS data.

The JHS Steering Committee approved the JHS Vanguard Center at RTRN DCC, executed the data-sharing agreement and authorized electronic transfer of the full JHS dataset, which is housed in a secured space on the DCC Portal. From 1 September 2009, the DCC assumed the role of JHSVC and started providing services designed to stimulate interest throughout the RTRN community to utilize the data available through the JHS in the development of scientific literature through scientific presentations, publications and ancillary studies.

## 3. Data-Sharing Approach Driven by RTRN DCC: User-Friendly Data-Sharing Practices

User-friendly data-sharing practice is defined as all forward activities for facilitating data reuse among the Network investigators by providing services that meet the individual investigator’s needs, which is driven by the Network Data Coordinating Center. The goal of the practice is to generate growth and to accelerate academic productivities for clients (Network investigators) through user-friendly professional data services. This can be accomplished by developing assertive marketing strategies that allow the Network investigators to quickly reach data and services and efficiently generate academic outcomes.

The data-sharing approach is, therefore, designed to expedite academic productivity through DCC’s customized services. Prior to preparing a manuscript proposal, investigators (data users) can seek the assistance of the DCC statisticians to test the feasibility of study hypotheses by means of simple statistical methods or relevant document review. Hence, investigators can start with only study ideas that can be investigated using JHS data. This approach increases the likelihood of the success of research projects. Investigators are supported by DCC staff experienced with JHS data, policies and guidelines. DCC provides consultation on fast processing, statistical methods and study design. This support expedites the communication between JHS and the investigator and shortens the manuscript processing time. Standard considerations, such as privacy and confidentiality, are sensitive issues in all data-sharing arrangements. Although this issue was well documented in the JHS policy and guidelines, it may be difficult for lay persons to understand it. The experienced DCC staff will help the data users handle this issue. DCC will also support cyber workgroups for key domains (e.g., cardiovascular, cancer) that bring scientists together to develop scientific strategies and set Network-related priorities. Table 1 presents the roles of DCC for each stage of manuscript development.

The expected benefits from this program included: (1) expediting translational research—JHS included a broad array of data, including the various genotype and phenotype factors that affect high blood pressure, heart disease, stroke, diabetes and other important cardiovascular diseases; this allows one to “translate” findings in basic research into medical practice and meaningful health outcomes; (2) facilitating JHS data access for RTRN investigators—the JHS Vanguard Center at RTRN is aimed to facilitate data sharing among investigators in the Network; therefore, relevant services provided by the DCC will facilitate data access for the Network investigators; (3) the use of comparative datasets—the JHS included only African American participants; therefore, it allows one to investigate racial differences in heart disease and risk factors impacting the disease by comparing to data from other heart studies, such as the Framingham Heart Study; and (4) expediting the initiation of ancillary studies, sub-studies and related projects—the experienced staff at DCC will help the Network investigators who want to develop an ancillary study, which will involve the acquisition of additional interviews, the examinations of study participants, the analysis of blood, urine, tissue or other samples or images previously collected that are not compiled as part of the standard JHS dataset [43].

**Table 1:** Roles of the Data Coordinating Center (DCC) per phase of research development.

Activities	DCC Services
Developing research ideas (or hypotheses)	Provide data information, such as questionnaires, summary data for major variables, variable list, *etc.*
Feasibility test	Conduct the preliminary analysis to determine the feasibility of the manuscript idea.
Manuscript proposal development	Provide consultation on statistical methods for the study. Write the section of the statistical analysis plan. Identify experts for the research topic. Submit the proposal on behalf of the principal investigator (PI). Liaison between the PI and JHS publication and presentation subcommittee (P&P). Respond to JHS P&P.
Data access	Prepare customized dataset once JHS P&P approval is obtained.
Data analysis and manuscript development	Conduct statistical analysis. Write results and part of the discussion section of the manuscript; liaison between PI and JHS P&P or the journal editor.

## 4. DCC Engagements to Facilitate Data Reuse

DCC as a Jackson Heart Study Vanguard Center has already been involved in various initiatives for data-sharing practices for Network investigators. The following is a description of how DCC has prepared for this initiative, what activities have been performed to promote data utility and how DCC has supported RTRN investigators in developing research through the JHS data.

### 4.1. Preparation of Services

Web search: In order for us to concentrate our marketing activities and efforts related to the data, a web search was conducted to identify potential users of the data. We reviewed the curriculum vitae and/or profile of faculty that were posted on the website of each member institution. The search identified 235 potential investigators within the Network in the research areas of cardiovascular disease, renal-related disease, diabetes, health science research, pulmonary disease, social psychology, nutrition, physical activity, obesity and social work. For the early stage of this data-sharing practice, promotional strategies focused on these identified potential data users.

Data-related materials: The materials that DCC prepared before launching the program included the survey form, format dictionary, variable list, manual of the process, data access guidelines and data book. This data book contained a comprehensive overview of the baseline examination on the prevalence of CVD and related major risk factors using the baseline data, including 5301 participants. The data book was intended to provide a useful reference for the potential data users when they start developing research questions and/or hypotheses. Graphics and charts were included for the presentation of the statistical output to increase readability for the users.

Data infrastructure: Data-related infrastructure included the data itself, data sharing tools and data analysis software. The DCC procured additional large datasets that were intended to be used for a comparison study with the Jackson Heart Study data. The large datasets included the National Health and Nutrition Examination Survey (NHANES), which is a program of studies designed to assess the health and nutritional status of adults and children in the United States where interviews and physical examinations are combined, and the Behavioral Risk Factor Surveillance System (BRFSS), which is the world’s largest, on-going telephone health survey system, tracking health conditions and risk behaviors in the United States yearly since 1984. All data were formatted into SAS (Statistical Analysis System) data format and shared through an access-regulated virtual workspace. A server-based SAS program allows collaborative investigators to remotely access analytical tools.

Website development: The RTRN JHSVC website [44] was developed and utilized as a tool for promoting the data and services and for sharing relevant information. The website is frequently updated to ensure relevant and timely information about the data and services. The content of the website includes: an overview of the Vanguard Center, the role of the DCC and its services in research development, JHS design and data components and data-related information (data dictionary, format dictionary, summary report, data workshop video clip, forms and manuals), service request and contact information.

### 4.2. Data Marketing Activities

Seminar series/workshops/consulting: The DCC has also organized various training programs, including webinar seminar series and statistical workshops and one-on-one statistical consulting at RCMI international symposia where most Network statisticians convened. The DCC provided four webinar seminars, three on-site workshops and on- and off-site consulting programs. More than 520 RTRN investigators and students have participated in these programs.

Outreach program: The purpose of outreach is to showcase DCC research capacity and to foster collaboration for future projects (publications and ancillary studies using Jackson Heart Study data) within the Network. The outreach tour was designed to stimulate the exchange of research ideas between the DCC and Network institutions in order to: (1) identify the statistical needs of RTRN investigators; (2) disseminate information about DCC expertise and capacity; and (3) identify future research projects that the DCC and the RTRN institution(s) can pursue jointly. A team of three senior statisticians from the DCC visited three geographic areas (comprising five RCMIs) from 23 May–10 June 2011 and interacted with a total of 129 investigators who participated in this program. The mobile program took “support” out into the scientific community and helped to minimize some of the barriers of communication between DCC and researchers. Table 2 represents the marketing activities of the DCC team.

**Table 2:** Promotional activities and venue.

Type	Topic	Method
Workshop	JHS data workshop as part of the RCMI Principal Investigator/Program Director (PI/PD) Meeting at the Mississippi e-Center/Jackson State University (March 2009)	On-site
Workshop	The 1st JHS Vanguard Center Workshop: Overview of JHS and Procedure & Guideline for Data Access (27 August 2009)	On-line
Workshop	The 2nd JHS Vanguard Center Workshop: Details of Jackson Heart Study Data (22 October 2009)	On-line
Workshop	Application of JHS Data to Biomedical Research	On-site
Mobile Workshop	JHS Vanguard Center workshop at Morehouse School of Medicine, Howard University, University of Puerto Rico, Universidad Central del Caribe, and Ponce School of Medicine (from 23 May–10 June 2011)	On-site
Booklet	Prepared 90-page booklet introducing JHS Vanguard Center and Jackson Heart Study; a total of 250 copies of booklet have been distributed to Network investigators and students	On-site and online
Website	Developed website describing an overview of the Jackson Heart Study Vanguard Center and how to access data and services	Online

### 4.3. Support of Data Reuse

Experienced staff-guided support: Three senior-level biostatisticians supported this engagement over the period of 2009–2013. The DCC staff has 40 years of combined experience in the Jackson Heart Study and has led/coauthored over 40 peer-reviewed scientific articles using JHS data. The team provided customized data services, such as providing data information, conducting feasibility tests, assisting with the preparation of manuscript proposals, conducting data analyses, and assisting with the development of ancillary studies and manuscripts.

**Figure 1:**
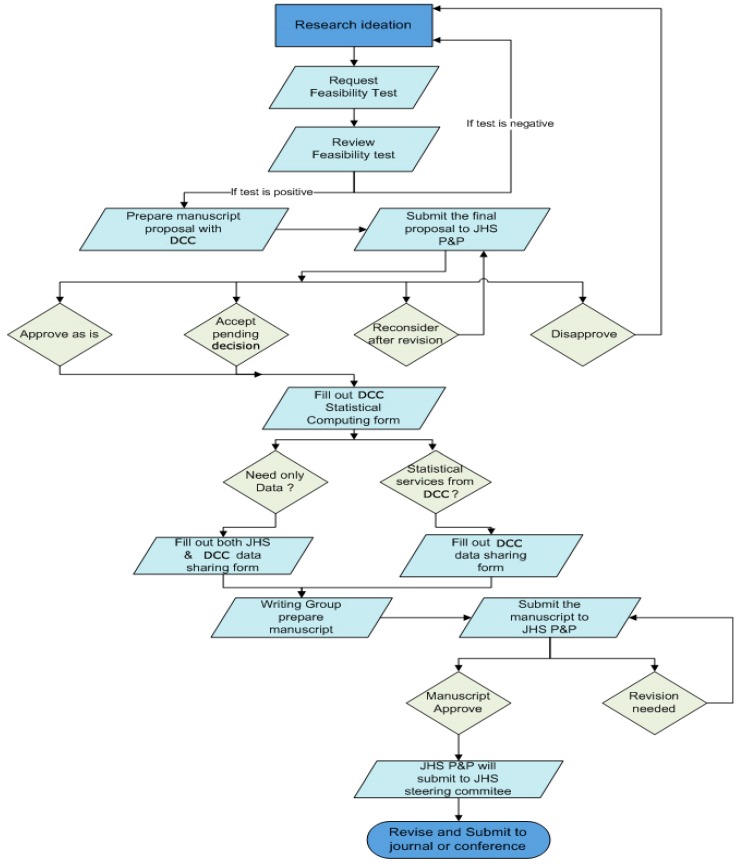
Process to develop research.

Procedure for manuscript development: The DCC developed the manuscript development procedure, which was combined with the Jackson Heart Study policy and procedure. It was important to include JHS rules and guidelines in the DCC procedure. The flow chart depicts the procedure for study development (Figure 1).

Feasibility test: The feasibility test may be the most unique aspect of the RTRN DCC data-sharing practices. Prior to preparing a manuscript proposal, which is a requirement to access the data, an investigator requests the DCC to test the feasibility of the study hypothesis that they generated with the information posted on the website. Based on the results of preliminary tests performed by DCC through material review and simple statistical analyses, investigators decide if the study idea could be supported by further analysis of the JHS data if it were not previously published or under analysis. Thus, RTRN investigators are able to start with only study idea that is feasible using JHS data. This, in turn, will minimize wasted effort and the time of the investigator and ensure a greater likelihood of success in developing and producing scientific manuscripts using JHS data. The RTRN investigators could request the feasibility test formally (by submitting the feasibility test request form posted on the website) or informally (sending an email or making a phone call). For the feasibility test, only limited statistical methods (e.g., chi-square for categorical data or *t*-test for continuous variables) were applied. More effort is required to answer the following questions through the review of relevant materials:

1. (Data feasibility) Are Jackson Heart Study data available for supporting the research idea?
2. (Methodology feasibility) Are quantitative methods (e.g., rare case data analysis or statistical data mining) available to conduct the data analysis?
3. (Expert feasibility) Does the research idea need help from any special expert(s) in the proposed area? Is there an expert within the Network who will help the research development?
4. (Topic duplication) Is the research idea duplicated with studies published and/or being worked on by JHS investigators and/or their collaborators?

Match-making: The RTRN Profiles and the profiles of 235 identified through the web search were used to build the manuscript development team. RTRN Profile is web-based tool to discover and use research and scholarly information about people and resources, which is managed by DCC and now includes approximately 1900 investigators.

Data-sharing agreement and authorship: Jointly with JHS and DCC, acknowledgement and adherence to the data-sharing agreement were mandated for any investigator accessing the JHS data. The investigator filled out the DCC statistical computing request form. If the investigator wanted data only, then he/she filled out both JHS and DCC data-sharing agreement forms. If the investigator wanted statistical services from DCC, then she/he submitted only the DCC data-sharing agreement form. The RTRN investigator included at least one DCC investigator or key personnel as co-authors, as appropriate, on any publications. By the Jackson Heart Study Publication and Presentation Guideline [45], at least one JHS investigator should be included as co-author on any publications. When possible, a general acknowledgement should be given to the JHS participants and data collection staff.

## 5. Discussions

As a Jackson Heart Study Vanguard Center, DCC has already been involved in various initiatives for collaboration with Network investigators to perform research activities within the Network. The collaboration strategy that the DCC has applied was to prepare an environment within which participants feel free to share ideas, information and work. Promoting the sharing Jackson Heart Study data was also one of the strategies to enhance collaboration among the Network investigators. Therefore, RTRN DCC data-sharing practices were designed as a user-friendly program in which the data user can freely contact DCC to discuss study ideas and how to access the data information. The DCC imposed only a minimum requirement on potential users in order for them to access data and receive services. The DCC has helped Network investigators generate scientific output by providing such customized services for secondary data use as preliminary analysis for feasibility, data querying, data workshops and idea-sharing meetings, profiling potential investigators for team building, providing data information and query tools through the website, helping develop manuscript proposals and providing data analysis for publications. More than 300 investigators/biostatisticians attended workshops and/or received booklets designed to help potential data users understand the data structure and how to access the data. The DCC provided three online and five onsite workshops, developed/distributed more than 250 copies of the booklet and posted instructions on the RTRN website to facilitate the data utility. Five related manuscript proposals, eleven presentations, seven published papers [46,47,48,49,50,51,52] and two R01 and two small (R03 and R21) grant proposals were supported.

Additionally, the JHS dataset has been routinely utilized as preliminary data to address questions raised in active research projects. For example, the JHS data were utilized for the Minority Health Genomics and Translational Research Bio-Repository Database Network (MH-GRID) case-control study to evaluate how relaxing inclusion and exclusion criteria would impact enrollment and statistical power. The JHS data and samples were harmonized with and integrated into the dataset of the MH-GRID hypertension use case study.

One of the important considerations in developing this program was to facilitate communication between DCC and the potential data users. Data workshops were designed for the scientists to gain an overview of the data in order to be able to develop appropriate research ideas. In addition, workshops for study design and statistical methods that would be applied to JHS data were to facilitate communication between DCC and Network scientists with a common language to identify major methodological issues in the active research idea and to make the statistical theories and approaches understandable and relevant to the scientist’s own field of interest.

The RTRN DCC practices have offered numerous advantages. First, unless investigators conduct the data analysis at their site, institutional review board (IRB) approval from the investigators’ institution may not be needed, since the DCC has obtained IRB approval for the JHS data use. Second, users usually begin writing manuscript proposals with limited information. They may find that their research idea is not feasible with JHS data after receiving reviewers’ comments from the JHS P&P Subcommittee. Feasibility tests done by experienced DCC staff allows data users to pursue those with a high possibility of success. Third, users can save the time that would be needed to understand the JHS Data Coordinating Center’s procedures and guidelines to access the data. The DCC staff who fully understand the JHS procedures and guidelines serve as liaisons between the JHS P&P Subcommittee and the users. Fourth, users can concentrate on writing papers or proposals, because the DCC provides total services from administrative to academic. Fifth, it is necessary for early-career scientists to network with those who can effectively translate and communicate the intricacies and value to various stakeholders [53]. Users can network with experts in the proposed research area through DCC match-making services.

Despite the advantages, it is important to note that a few challenges can affect the efficiency of the practices and the utility of data reuse. First, personnel for engaging in this aggressive data sharing is limited by the resources at the DCC. The limited personnel reduces supporting activities, and the reduced activities may affect the outcomes. In reality, most academic outcomes resulted from supporting activities done during the initial period of the practice when a significant number of supporting staff performed the intensive promotional activities. The main duties of the DCC staff involved in this practice were to support the Network investigator-initiated clinical trials and to develop the statistical capacities of the Network. It might be an additional burden for them to engage in this practice without such support. To be sustainable, it may be important to require close articulation with cyber workgroups to ensure the inclusion of DCC team members in research grant proposals. Activities related to data sharing consist of education, training and support. It is crucial to budget these activities when developing grant proposals for data sharing, since these costs are underestimated and under-awarded [54]. In some settings, a fee-for-service mechanism could be used, but might be a barrier for early-career scientists, who are most likely to need the services. Although DCC might not be able to support additional personnel for the Vanguard Center, biostatisticians can be solicited from the Network. Because there are limited collaborators who are experienced in the study data, a well-designed training program for voluntary collaborators may be necessary.

Second, JHS data are local, but the Network is national. Thus, some investigators may not feel the data are applicable and/representative of their community and not feel compelled to take advantage of this resource. In order to pique the interest of the researchers from broader geographical areas, Jackson Heart Study data need to be harmonized with and integrated into the other local-based heart study datasets, such as the Kohala Health Research Project (formerly known as the Native Hawaiian Health Research Project), the Mississippi Delta Cardiovascular Health Examination Survey and the Puerto Rico Heart Health Program. All of these studies were conducted in the area where the member institutions are located.

Third, heart disease is only one of the network concerns, and even with the workshops, it may not have been explicit that the resources in the JHS extend far beyond heart disease and may have relevance to a broader segment of the RTRN constituency. A more complete description of the JHS dataset, which includes how its utility extends beyond heart disease, would capture a wider audience, including established investigators. It may be also important to broaden the variety of datasets, which will cover topics other than heart disease. This study focused on the DCC data-sharing practices, which were performed before a broad-based RTRN data-sharing policy was prepared. It was only recently that the RTRN prepared a comprehensive data-sharing policy. Therefore, to provide services for an enhanced variety of datasets, the current JHSVC data-sharing practices driven by the DCC may need to be revised according to the newly-developed RTRN data-sharing policy.

Fourth, since the main users were scientists with modest or substantial experience in data use, limited services were actually utilized for this data-sharing practice. This program was designed based on an assumption that the main users would be faculty with limited experience in accessing large secondary data. Most users were, however, experienced in large databases, such as the Jackson Heart Study data. Several had at least a couple of secondary database publications. DCC provided only limited services for those users (e.g., statistical support, part of the writing team, *etc.*). It was estimated that approximately 30% among the total registrants in the RTRN Profile were early-career investigators [55]. Therefore, to optimize the utility of data, promotional activities should be intensified for these researchers who are expected to be eager to create academic productivity through the use of secondary data for obtaining the next academic ranking. When performing promotional activities for this target group, it is important to consider the following issues: (1) although webinars, workshops and brochures/booklets were effective during the initial phase of the program, available new marketing tools, such as social media and the use of mobile apps, should be applied to the early-career investigators, which might have a greater impact on the target group; (2) as with any promotion, there will be early adopters and then those that wait to see the successes or challenges that these individuals have before they themselves decide to participate; the successes should be widely publicized to satisfy the curiosity of this group; (3) an overall long-term strategy must be developed that could include “review” and “update” every 2–3 years to interest new investigators entering academia and keep the more experienced investigator interested.

Fifth, it may be difficult to generalize the results of the study globally because all network participants were in the U.S. Despite that limitation, our study introduced a user-friendly data-sharing practice within a U.S. research network that includes scientists from Hawaii to Puerto Rico and across multiple distinct institutional cultures representing different local languages and cultures. A data-sharing research network that represents users that differ substantively by region, including culture and primary language, makes it more likely to have the most generalizability afforded by a single-nation study.

Last, though it is important to obtain feedback from the participating scientists regarding what they found useful, this program does not include any measures to judge the success of the various interventions nor provide any sort of quantitative marker of success. Therefore, monitoring its success on a regular basis should be planned at the beginning of the program implementation. Ongoing monitoring has been used to detect a change in outcomes so that the process can be examined, reinforcing beneficial practices and eliminating factors that degrade performance [56,57]. The proposed performance and process indicators and benchmarks will be used to accomplish this end.

## 6. Conclusions 

Despite some limitations, our study suggested that DCC-customized services enhanced the accessibility of JHS data and its utility by RTRN researchers and helped to achieve the principal JHSVC goal of increasing scientific productivity and expanding the dissemination of the JHS findings. However, a lack of a dedicated DCC personnel budget for JHS support may have become a barrier for the long-term effectiveness of the collaboration. Productivity could likely be further increased significantly if additional resources are invested in the initiative. Other similar models of networking collaboration with highly relevant external projects should be prepared to dedicate resources to projected activities in order to maximize the likelihood of success.
